# Determination and Monitoring of Quality Parameters: A Detailed Study of Optical Elements of a Lens-Based Raman Spectrometer

**DOI:** 10.1177/00037028211055148

**Published:** 2021-10-29

**Authors:** Ashutosh Mukherjee, Anita Lorenz, Marc Brecht

**Affiliations:** 1 Center for Process Analysis and Technology (PA&T), School of Applied Chemistry, 64332 Reutlingen University, Reutlingen, Germany; 2 Reutlingen Research Institute (RRI), 64332 Reutlingen University, Reutlingen, Germany; 3 Institute of Physical and Theoretical Chemistry, University of Tübingen, Tübingen, Germany

**Keywords:** Raman spectroscopy, principle of Raman spectrometer, spectral resolution, full width half-maximum, FWHM

## Abstract

A lens-based Raman spectrometer is characterized by studying the optical elements in the optical path and we study the measure of aberration–diffraction effects. This is achieved by measuring the spectral resolution (SR) thus encompassing almost all optical elements of a spectrometer that are mostly responsible for such effects. An equation for SR is used to determine the quality factor *Q* which measures aberration/diffraction effects occurring in a spectrometer. We show how the quality factor changes with different spectrometer parameters such as grating groove density, the wavelength of excitation, pinhole width, charge-coupled device (CCD) pixel density, etc. This work provides an insight into the quality of a spectrometer and helps to monitor the performance of the spectrometer over a certain period. Commercially available spectrometers or home-built spectrometers are prone to misalignment in optical elements and can benefit from this work that allows maintaining the overall quality of the setup. Performing such experiments over a period helps to minimize the aberration/diffraction effects occurring as a result of time and maintaining the quality of measurements.

## Graphical abstract

**Graphical Abstract:**
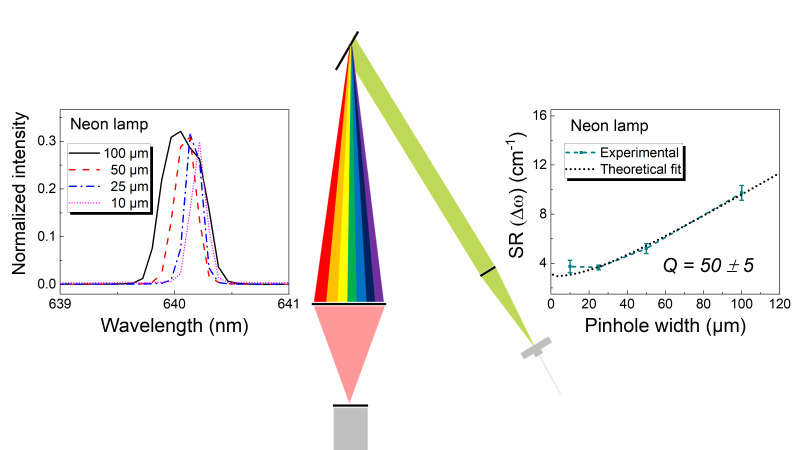

## Introduction

Raman spectroscopy is one of the most powerful techniques in identifying chemical compositions of a sample through designated peak positions also known as the chemical signature/fingerprint of a material.^1^ Apart from material identification, typical Raman spectra contain rich information about specific properties such as crystallinity, doping, stresses, etc. For example, the width of a peak may indicate the crystallinity of a material,^2,3^ intensity and intensity ratios determine the concentration (per unit area exposed to the laser) and relative quantities of materials present,^4^ a peak shift from its signature position may determine whether the material is stressed/strained,^5–9^ doped,^10–13^ or in an excited state, e.g., at elevated temperature.^13–16^ However, not always are these cases prevalent and often there are peak shifts or broadened peaks irrespective of their origin, and generally such details are ignored as long as they do not fit the aim of the experiment. If the shift of the peak of interest is within the permissible limits of the spectral resolution (SR), then it is critical to commemorate the origin of the shift to the factors concerning the strain, doping, crystallinity, temperatures, etc. Therefore, it is of utmost importance to study the spectrometer properties before concluding any critical analysis of any material under investigation.

A good spectrometer design (SD) for Raman instruments in general, and Raman microscopes in particular, can solve the problem and the SD is one of the most important factors in assessing the quality of a spectrometer or measurement. The basis of good SD in a Raman instrument (or spectrometer) lies in the effectiveness of the optical elements in the optical path to form an image of the entrance slit at the exit plane with the wavelengths involved in the excitation source. This can be seen in its optimum signal-to-noise ratio and in the spectral resolution of the measured Raman spectra, in the depth resolution of the microscope, etc. The elements on which the aforesaid qualities of spectrometer depend are the excitation source, objective (magnification/numerical aperture), grating groove density, slit width/pinhole size, focal length, system magnification, pixels of the charge-coupled device (CCD), and other aberration/diffraction effects occurring due to optical elements in the setup like lenses and mirrors. For a good spectrometer, an optimum combination of all the aforementioned factors and their interaction must be considered and optimized.

However, the challenge to deal and consecutively compensate for aberrations/diffractions is persistent. In some of the commercial Raman microscopes, the optical path usually is handled with mechanical interfaces such as beam steering motors, motors for grating rotation, lens adjustments, slit opening, beam splitters, mirrors, etc*.* Such interfaces undergo creep or hysteresis, and over a certain period the motors are not in the same position as they should have been during the manufacturing and calibration of the machine.

This leads to discrepancies in the experimental data, which must be tracked and investigated to maintain the optimal quality of the spectrometer. These discrepancies can be eliminated if the optical path is thoroughly calibrated each time, which can be a challenge for commercial spectrometers. An optical beam is very sensitive to any changes of optical elements in the beam path. These changes can lead to spherical, chromatic aberrations, diffractions, beam deflections, reflections, etc. Therefore, the elimination of such effects is an important challenge in optical spectroscopy. A typical SD used in a Raman instrument is an uncrossed Czerny–Turner configuration, consisting of two concave mirrors and one planar diffraction grating and has proved to be the most suitable configuration for Raman spectrometer.^17–21^

It is important to study the Raman spectrometer without manipulating the elements in the optical path. To study every element in the optical path is time consuming and is a challenge by itself. In this work, we describe how the quality of a spectrometer can be studied in detail in a commercial Raman microscope comprising all elements in an optical path. This work has been inspired by the previous work of Liu and Berg^21^ where they analyzed an uncrossed Czerny–Turner spectrometer by measuring its SR. Further, they determined the aberration/diffraction correction factor for their setup.^21^ Conducting the described procedure over a certain period will help to maintain the quality of a setup and, in case of problems, to be able to identify their origin and solve them accordingly.

## Mathematical Background

Studying the SR of the spectrometer will include a major portion of the elements in a Raman instrument such as the grating groove density, focal length, lenses/mirrors, CCD pixels, and pixel size.

### Spectral Resolution

To determine the SR with the available options, Liu and Berg^21^ combined all the factors that influence SR into one expression, the derivation of this equation is explained in detail in previous studies.^20,21^ The advantage of this equation is that it shows the dependence of SR under different optical configurations. The factors affecting SR can be summarized in Eq. 1:

$$\begin{array}{c} \Delta \omega [\omega_{L},\omega_{R},G,f_{2},\alpha ,b_{\mathrm{img}}]\;=(\omega_{L}-\omega_{R})2\cdot \frac{b_{\mathrm{img}}}{G\cdot f_{2}}\;\;\cdot \text{cos}\{\arcsin(\frac{G}{2\cdot (\omega_{L}-\omega_{R})\cdot \text{cos}(\frac{\alpha }{2})})+\frac{\alpha }{2}\}\;\;\;(1) \end{array}$$

in which Δω is SR, $\omega_{\text{L}}$ is the absolute wavelength of the excitation source, $\omega_{R}$ is the Raman wavenumber shift of the desired peak under investigation, *G* is the grating groove density per µm, the groove’s distance can be calculated as *d *= 1/*G* (µm), *f*_2_ is the focal length of the system (distance from a second Czerny–Turner lens to the CCD), α is the angle between the incident and diffracted beam on the grating, also known as “include angle”. The calculation for this angle is shown in detail in the Supplemental Material, *b_img_* is the full width half-maximum (FWHM) of the entrance slit or pinole. FWHM of the entrance pinhole/slit is limited by aberration/diffraction effects. To take this into account, an empirical relationship^21^ between *b_img_* and the entrance pinhole/slit width *b_ent_* is given by Eq. 2:

$$b_{\mathrm{img}}=M\cdot \sqrt{(b_{\mathrm{ent}}{)}^{2}+(b_{\mathrm{limit}}{)}^{2}}\;\;\;\;\;\;\;\;\;\;(2)$$

where *M* is the system magnification and *b_limit_* is the smallest value possible of FWHM of the pinhole/slit width.

When *M* = 1, *b_limit_* is given by

$$b_{\mathrm{limit}}=\frac{Q}{\left(\omega_{L}-\omega_{R}\right)}\;\;\;\;\;\;\;\;\;\;\;\;\;\;\;\;(3)$$

Here, Q, the so-called quality factor is approximated as a constant for a spectrometer with a specific design.^21^ It is calculated by best fitting the experimental results to the theoretically derived values using Eq. 1. The resulting curve for SR as a function of pinhole/slit width is a hyperbolic curve which will be discussed later in the results section.

Liu and Berg^21^ used an uncrossed Czerny–Turner monochromator (Renishaw) where they compared spectral resolutions of two different lasers and different gratings and measured the correction factor of their setup.^21^ They have attributed “quality factor *Q*” as diffraction and aberration correction factor (DACF) or A. In this paper, we deal in detail with the quality factor *Q* and with the parameters that influence *Q*. It is important to study how it variates if different parameters in Eq. 1 change, e.g., the excitation wavelength, the grating, etc.

## Materials and Methods

**Figure 1.**
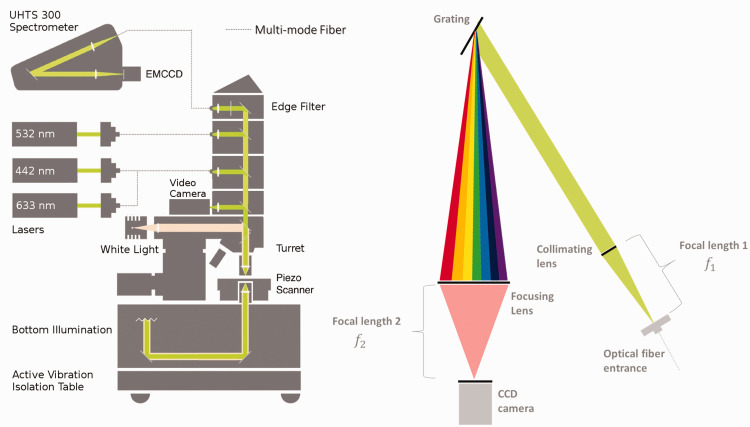
Schematic of the optical path of WITec alpha 300RAandS. Left: Beam path of the confocal Raman microscope. Right: Beam path inside the spectrometer.

A single crystalline diamond was used as a sample with a characteristic Raman peak at 1332.4 cm^−1^ and a true Raman FWHM of 1.2 cm^−1^.^22^ For atomic emission spectra, a calibration neon lamp from Kaiser Electronics was used. A commercial Raman microscope from WITec (Alpha300 RAandS) was used and a detailed optical path is sketched in Fig. 1. Raman spectra were obtained using an air objective (Carl Zeiss; EC Epiplan-Neofluar DIC M27, 100×, NA = 0.90). This system is equipped with a lens-based UHTS 300 spectrometer connected using a multimode optical fiber and thermoelectric cooled CCD and electron multiplying CCD (EMCCD, Andor DU970N-BV). The CCD and EMCCD is a back-illuminated CCD with 1600 × 200 pixels and each pixel is 16 µm × 16 µm. The used multimode fibers had different diameters that transported the scattered signal to the spectrometer. Thus, the diameter of these fibers determines the size of the pinhole. They were 10 µm, 25 µm, 50 µm, and 100 µm all with a NA = 0.12. For excitation, a diode laser (532 nm) with a nominal output power of 40 mW, a helium–neon laser (633 nm) with an output power of 25 mW, and a helium–cadmium laser (442 nm) with an output power of 25 mW were used. Data processing was done using Control Project Plus 5.0 software provided by WITec and all experiments were carried out at ambient conditions.

## Results and Discussion

Equation 1 shows that SR ( Δω ) depends on factors that can be classified into three types: (i) instrument-specific, (ii) experiment-specific, and (iii) sample-specific parameters. The instrument-specific parameters are those parameters that are fixed during manufacturing like system magnification, incident/diffraction angle, aberration/diffraction effects, focal length, and CCD pixels. For a commercially available Raman microscope, instrument-specific parameters cannot easily be changed since the complete optical path is calibrated and fixed precisely. Experiment-specific parameters are specific to a certain experiment/measurement that can sometimes be changed depending upon the available options. These include the grating groove density (*G*), excitation source wavelength (ω*_L_*), pinhole/slit widths. Sample-specific parameters are those parameters that completely depend on the sample under an investigation like the Raman peak positions of the sample (ω*_R_*). Out of all these experiment-specific parameters, only pinhole widths can be changed to reliably determine the SR.

**Figure 2.**
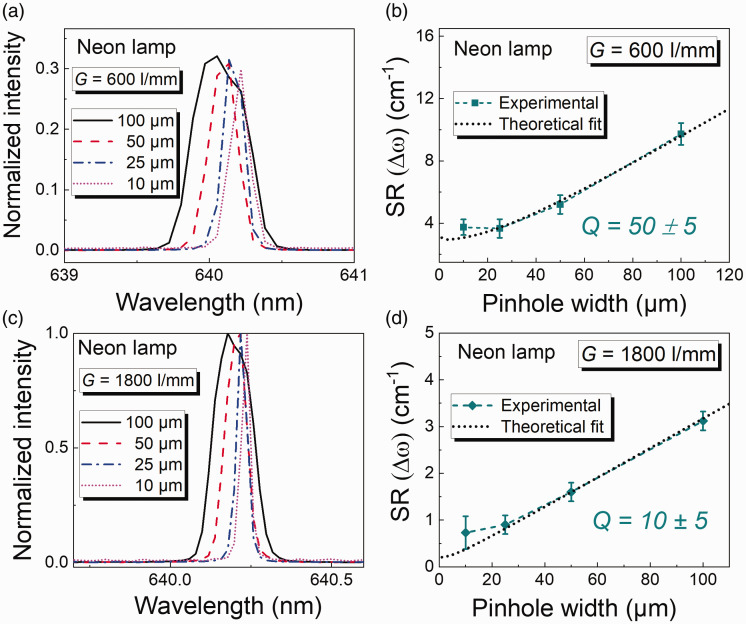
(a) Neon lamp spectra at 640.2 nm measured with different pinhole widths and a grating of 600 lines/mm, (b) experimentally measured and theoretically calculated FWHM plotted as a function of pinhole width and a grating of 600 lines/mm using Eq. 1, (c) neon lamp spectra measured with different pinhole widths and a grating of 1800 lines/mm, and (d) experimentally measured and theoretically calculated FWHM plotted as a function of pinhole width and a grating of 1800 lines/mm using Eq. 1.

We first studied the case where ω*_R_* = 0, by using atomic emission lines consisting of a single wavelength that is measured as a function of pinhole width. This reveals the true potential and the limit of determining the SR. For this reason, these lamps that are emitting a single frequency are generally used for calibration of the setups.^23,24^ Here, a neon lamp is used. The lamp is placed directly under the 100× (NA = 0.9) objective so that the light follows the optical path like from any other sample. For the experiment, the emission line at 640.2 nm with a natural line width of 0.0001 nm was chosen and the results are shown in Fig. 2.^25,26^

Figures 2a and 2c show the emission line of the neon lamp at 640.2 nm depending on available pinhole widths for both the gratings. At smaller pinhole widths (10 µm, 25 µm) the emission line is sharper compared to the emission lines at larger widths (50 µm, 100 µm). This can be explained by the fact that with larger pinhole diameters the point source becomes broader resulting in a broadened line width. It can be observed that there is a gradual shift observed in the emission line position starting from pinhole width 100 µm to 10 µm. Although the line under investigation has a very narrow linewidth, it is significantly broadened when measured with 100 µm pinhole width (broad top-hat function). The emission lines positions observed in the Figs. 2a and 2c range from 640.17 nm to 640.2 nm. If calculated, the Raman shift would be Δω*_L_* ∼ 2–3 cm^−1^, all other measurements with smaller pinhole widths lie in this range. This small value does not affect our calculated results later on and hence can be neglected. Each of the spectra in both Figs. 2a and 2c looks edged and does not have a typical Lorentzian line shape. This is because of the CCD pixel widths in which the density of CCD pixels is not enough to give a perfect line shape to the spectra. Figures 2b and 2d show the experimentally measured FWHM plotted along with the theoretically calculated SR using Eq. 1. Substituting ω*_L_* = 640.2 nm and ω*_R_* = 0 in Eq. 1, yields for the 1800 l/mm grating, a fitting value of *Q* that is 10 ± 5 and for the 600 l/mm grating, the value of *Q* that fits best is 50 ± 5. These values of *Q* give the best fit with experimentally measured results.

For both gratings, experimentally measured data fit very well to the theoretically calculated values and deviates only for the smallest pinhole widths (10 µm). This disagreement is because of two reasons: first, at smaller pinhole widths the effects of aberration/diffraction become more prominent, and second, the width of the CCD pixels yielding an artificial broadening. For samples containing almost single frequencies (in this case a neon lamp), the constituent dispersion does not cause as many aberration/diffraction effects. Thus, this disagreement can arise due to the width of the CCD pixels and this agrees with previously published literature for smaller slit widths.^21^

**Figure 3.**
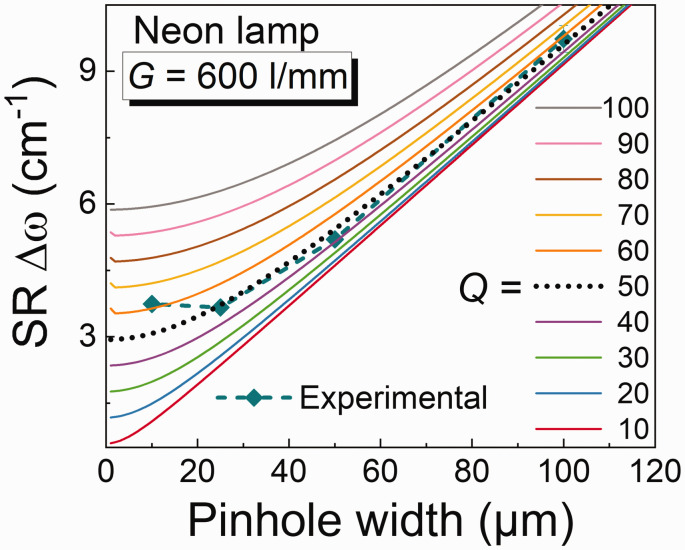
Different values of Q simulated to the experimentally measured results from Fig. 2.

To interpret these results, it is important to better understand *Q*. If the value of *Q* is changed then the agreements between the experimentally measured values and theoretically calculated values also change. An example of this is shown in Fig. 3.

Figure 3 shows the various values of *Q* ranging from 10 to 100 fitted to the experimentally measured data shown in Fig. 2b. It can be seen that for *Q* = 50, it gives the best theoretical fit for the experimentally measured data using Eq. 1. It can be observed that at higher values of *Q* the SR gets worse, and the effect of the pinhole size becomes smaller. Therefore, the quality factor *Q* can be directly related and defined by aberrations–diffraction effects occurring in the setup due to misalignment in the optical path. The higher the *Q* value, the higher the aberration/diffractions leading to a lower SR and vice versa. However, SR also depends on the excitation wavelength, thus a larger wavelength chosen would give a better SR. Such a relation is plotted in the Fig. S2a, (Supplemental Material) for both the gratings and is also known from the literature.^21^

**Figure 4.**
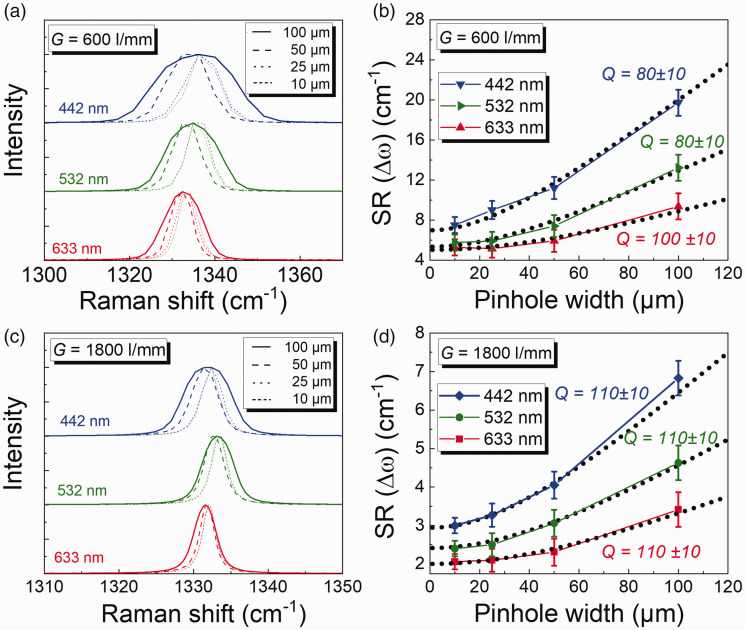
(a) Diamond spectra measured with 600 lines/mm grating with three excitation wavelengths, (b) measured FWHM of diamond spectra at 1332.4 cm^−1^ for 600 lines/mm measured with pinhole widths 10 µm, 25 µm, 50 µm, and 100 µm and different excitation wavelengths 442 nm (blue symbols), 532 nm (green symbols), and 633 nm (red symbols compared to theoretical calculations as per Eq. 1; black dashed lines), (c) diamond spectra measured with 1800 lines/mm grating with three excitation wavelengths, and (d) measured FWHM of diamond spectra at 1332.4 cm^−1^ for 1800 lines/mm measured with pinhole widths 10 µm, 25 µm, 50 µm, and 100 µm and different excitation wavelengths 442 nm (blue symbols), 532 nm (green symbols), and 633 nm (red symbols) compared to theoretical calculations per Eq. 1 (black dashed lines).

Now, in the case of ω*_R_* ≠ 0, by using a Raman scatterer (diamond, in this case) for three different lasers, values of ω_L_ (442 nm, 532 nm, 633 nm) are investigated. Diamond has a peak position at 1332.4 cm^−1^ corresponding to the triply degenerated vibration of the two Bravais lattices of the carbon atoms and with a natural bandwidth of 1.2 cm^−1^.^22,27,28^ The neon lamp used before has a natural line width that is much narrower than the natural linewidth of diamond and this difference must be considered. An analysis of the Raman spectrum of diamond with the same methodology is shown in Fig. 4.

Figures 4a and 4c show the spectra of a diamond measured at different pinhole widths for both the gratings and three excitation sources. The spectra do not have a typical Voigt line shape due to the limitation in the CCD pixel width as discussed earlier. However, it can be seen that the SR improves with larger wavelengths. It can also be observed that with different wavelengths of excitation, the peak position of the diamond (at 1332.4 cm^−1^) also shifts. However, this peak position shift does not affect our results later and hence can be ignored but must be considered where wavenumber accuracy plays a role. Figure 4b shows the results of the experiment with a 600 l/mm grating for three different values of ω*_L_*. The natural linewidth of a diamond is 1.2 cm^−1^ and is within the CCD resolution, so it is much larger than the natural linewidth of the neon lamp.

As a consequence, there is almost no deviation between the experimental value and the theoretical fit as can be seen in Figs. 4b and 4d for a pinhole width of 10 µm. The *Q* value for the 633 nm excitation source (red curve) obtained is 100 ± 10, for 532 nm (green curve) and 442 nm (blue curve) are 80 ± 10. Figure 4d shows the results of the experiment with an 1800 l/mm grating, for three different values of ω*_L_*. It is found that the experimentally measured values agree with the theoretically calculated values, and *Q* is 110 ± 10 for all three excitation sources.

According to Eq. 1, SR is inversely proportional to ω*_L_* and ω*_R_*, these are the two main reasons why the numeric value of SR varies here. The experiments are shown in Figs. 2 and 4 differ only in ω*_L_* and ω*_R_*. The experiments shown in Fig. 2 have only an excitation emission line ω*_L_* = 640.2 nm and ω*_R_* = 0. The natural linewidth is narrow and, hence, the SR is low. This results in the *Q* value of 10 ± 5 (for 600 l/mm grating) and 50 ± 5 (for 1800 l/mm grating). The diamond experiments as shown in Fig. 4 have three different excitation sources ω*_L_* = 442 nm, 532 nm, and 633 nm, and ω*_R_* = 1332 cm^−1^. Here, the natural linewidth of the sample depends also on $\omega_{\text{R}}$ resulting in broader line. This results in SR being higher than for neon emission lines and hence the value of *Q* is higher.

The value of *Q* depends on ω*_L_* and ω*_R_* and will change if any one of them varies. For example, if ω*_R_* is further increased to 3000 cm^−1^, this would result in a lower natural line width of the peak resulting in a different value of SR and, hence, a different value of *Q*. Such a dependence of SR as a function of ω*_R_* is shown in the Fig. S2b (Supplemental Material) for three excitation wavelengths and two gratings.

The value of *Q* can be manipulated to find the best fit between experimental and theoretical results. In Fig. 4b, it can be observed the SR are approximately similar with a grating 600 l/mm at smaller pinhole widths (10 µm/25 µm), for excitation sources 532 nm (green curve) and 633 nm (red curve). The deviation occurs only from a pinhole width of 50 µm. This is because with $\omega_{L}$ values being so close to each other (532 nm and 633 nm) the SR cannot be properly differentiated due to the lack of CCD pixel resolution for this grating. With 1800 l/mm grating as shown in Fig. 4c, it is worthwhile to note that at 10 µm pinhole width using a 633 nm excitation source the SR obtained is approximately 2 cm^−1^. This FWHM of the measured peak is very close to the true bandwidth of the diamond that is 1.2 cm^−1^. For a real-life sample if the combination of larger ω*_L_* and smaller pinhole width, the SR can be close to the natural bandwidth of the sample. Overall, the experiments shown prove that the quality of the spectrometer used is good.

Our results differ slightly from the previously published literature by Liu and Berg*.*^21^ We believe the principal reason for this is the different designs of the spectrometer. They characterized the SR of an uncrossed Czerny–Turner spectrometer (designed by Renishaw) and obtained a correction factor *A* (in their paper) to be 100. In their work *A* remains the same for the diamond sample as well as for a mercury lamp and does not change with a change in grating or excitation source. Nevertheless, the SR of the mercury lamp was comparable to the SR of our diamond sample at the 532 nm excitation source.

Spectral resolution as per Eq. 1 should also depend on the pixel density of the CCD camera and this can also change with the readout mode of the CCD camera. Experiments were performed keeping this in mind and changing the binning of the CCD camera resulting in no change in the SR. This is because the multimode fiber and its core diameter act as the entrance aperture of the spectrometer, eliminating the necessity of an additional slit system at the exit of the spectrograph. An additional slit system would cause the dispersed light to fall only on certain defined pixels of the CCD which might affect SR. In our case, the dispersed light covers the entire CCD chip thus the effect of SR only depends on the core diameter size of the multimode fiber.

## Conclusion

The spectral resolution (SR) of a lens-based Raman spectrometer (WITec alpha300 RAandS) is influenced by instrument-specific, experiment-specific, and sample-specific parameters. To determine the SR, two cases were considered from Eq. 1, first ω*_L_ *≠ 0: ω*_R_* = 0, and second ω*_L_* ≠ 0: ω*_R_* ≠ 0. For the first case (ω*_L_* ≠ 0; ω*_R_* = 0), a neon lamp was used, and the SR of an emission line at ω*_L_* = 640.2 nm was characterized. The narrow natural line width of an atomic emission line depicts the best possible SR attainable by a spectrometer. It was found that with the best possible SR was with 1800 l/mm grating and 10 µm pinhole width the attainable SR can approach values near or below 1 cm^−1^. The *Q* value or the quality factor depends on the agreement between the experimentally obtained and theoretically calculated results. The best-simulated agreement between them gives the value of *Q*. It was found out that for 600 l/mm grating, the *Q* value obtained was 50 ± 5 and for 1800 l/mm the *Q* value obtained was 10 ± 5.

For the second case ( $\omega_{\text{L}}\neq 0\vdots \omega_{\text{R}}\neq 0$ ), a diamond sample was used (ω*_R_* = 1332.4 cm^−1^) and three excitation wavelengths ω*_L_* (442 nm, 532 nm, and 633 nm). It was found that the best obtained SR was for ω*_L_* = 633 nm and a pinhole width of 10 µm resulting in a value of Δω*_R_* = 2 cm^−1^. This value of SR is very close to the natural FWHM of diamond (Δω*_R_* = 1.2 cm^−1^), and hence proves the quality of the spectrometer. The *Q* value obtained for 600 l/mm grating is 80 ± 10 (for 442 nm and 532 nm) and 100 ± 10 (for 633 nm). Whereas for 1800 l/mm, the *Q* value obtained is 110 ± 10 for all excitation wavelengths. In these experiments the value of *Q* is higher than for neon lamp experiments since the natural linewidth of a diamond is considerably higher than the neon lamp emission line.

Determining the quality factor *Q* and the SR of a spectrometer is straightforward with standard samples as shown here. It is therefore advisable to routinely perform the described protocol to monitor the quality of the setup and thus ensure the quality of the results in the long run.

## Supplemental Material

sj-pdf-1-asp-10.1177_00037028211055148 - Supplemental material for Determination and Monitoring of Quality Parameters: A Detailed Study of Optical Elements of a Lens-Based Raman Spectrometer Click here for additional data file. Supplemental material, sj-pdf-1-asp-10.1177_00037028211055148 for Determination and Monitoring of Quality Parameters: A Detailed Study of Optical Elements of a Lens-Based Raman Spectrometer by Ashutosh Mukherjee, Anita Lorenz, Marc Brecht in Applied Spectroscopy
