# Supplementary material for: Determination and Monitoring of Quality Parameters: A Detailed Study of Optical Elements of a Lens-Based Raman Spectrometer
Source: Appl Spectrosc. 2021 Oct 29;76(2):199–206. doi: 10.1177/00037028211055148 (PMC8832553; doi:10.1177/00037028211055148)
Supplement: sj-pdf-1-asp-10.1177_00037028211055148 - Supplemental material for Determination and Monitoring of Quality Parameters: A Detailed Study of Optical Elements of a Lens-Based Raman Spectrometer [file sj-pdf-1-asp-10.1177_00037028211055148.pdf]

Supplemental Material

Determination and Monitoring of Quality Parameters: A Detailed Study of Optical Elements of a Lens-Based Raman Spectrometer

Ashutosh Mukherjee[1,2,3], Anita Lorenz[1,2], and Marc Brecht[1,2,3]*

[1]Center for Process Analysis and Technology (PA&T), School of Applied Chemistry, Reutlingen University, Alteburgstrasse 150, 72762 Reutlingen, Germany

[2]Reutlingen Research Institute (RRI), Reutlingen University, Alteburgstrasse 150, 72762 Reutlingen, Germany

[3]Institute of Physical and Theoretical Chemistry, University of Tübingen, Auf der Morgenstelle 18, 72076 Tübingen, Germany

*Corresponding author email: marc.brecht@reutlingen-university.de

Calculation of Incident + Diffraction Angle

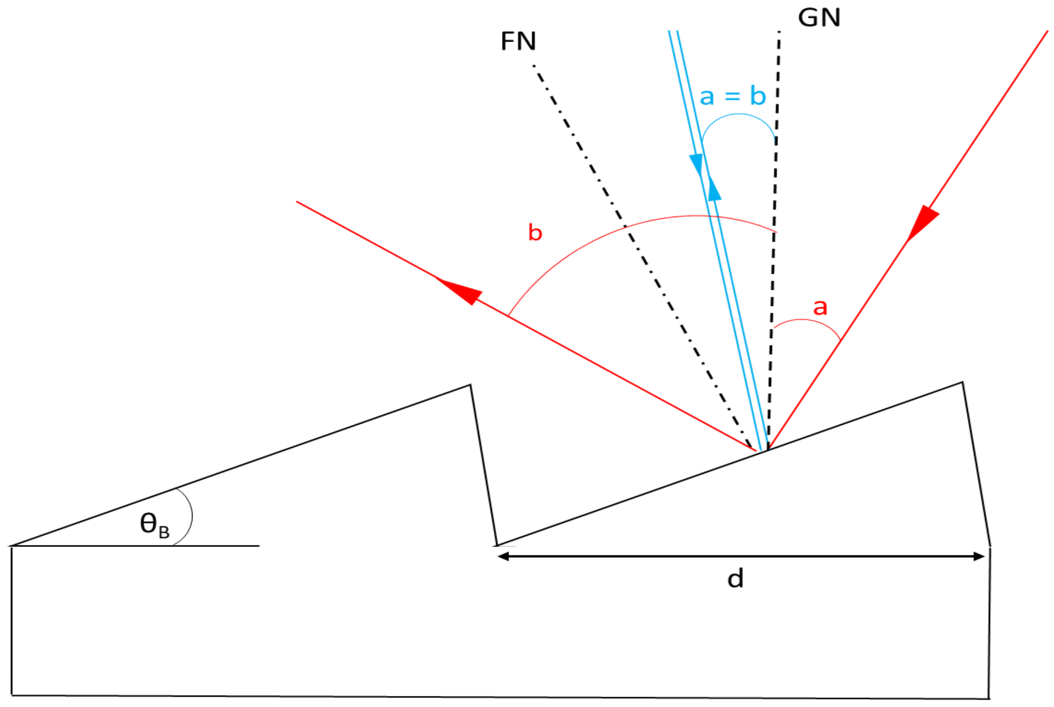

Figure S1. Schematics of blazed grating: The general case is shown with red rays and the Littrow configuration is shown with blue rays. GN is grating normal: the normal drawn to the plane of grating and FN is facet normal: the normal drawn to the facet of the grating.

The grating equation is given by Loewen and Popov:[1]

$$m\lambda = d(sina + sinb) \quad (1)$$

Manufacturing specification states that the two gratings (1800l/mm and 600 l/mm) are configured at a Blaze wavelength of 500 nm. Blaze wavelength λ_B is given by[1]

$$\lambda_B = \frac{2d}{m} sin\theta_B \quad (2)$$

At Blaze configuration,

$$\theta_B = \frac{a+b}{2} \quad (3)$$

where, a is the incident angle to GN and b is diffracted angle to GN, θ_B is the Blaze angle it is the angle between the face of the groove and the plane of the grating, m is the diffraction order and d is the grating groove density.

Solving for θ_B from Eq. 2 for both gratings,

$$\theta_B\ for\ 1800 \frac{l}{\mathrm{mm}} = 27.03° \quad (4)$$

$$\theta_B\ for\ 600 \frac{l}{\mathrm{mm}} = 8.66° \quad (5)$$

Now, let us assume that we have the Blaze condition for maximum efficiency that is the Littrow configuration. In this configuration, the incident and the diffracted angle are in the same plane that is $a = b$.

At Littrow configuration,

$$\theta_B = \frac{a+\beta}{2} = \frac{2a}{2} = a = b \quad (6)$$

This suggests either incident or diffracted angle has to be 27.03° for 1800 l/mm or 8.66° for 600 l/mm.

Substituting, $a\ or\ b$ in the grating equation (1) and solving for the remaining angle gives for say $\lambda = 633$ nm

$$b\ for\ 1800\frac{l}{mm} = 44.14°$$

$$b\ for\ 600\frac{l}{mm} = 13.34°$$

Now, at 633 nm,

$$\alpha\ at\ 1800\frac{l}{mm} = 90 - (a + b) = 90 - (27.03° + 44.14°)\ = 71.17°$$

$$\alpha\ at\ 600\frac{l}{mm} = \ 90 - (a + b) = 90 - (8.66° + 13.34°)\ \ = 22.00°$$

Similarly, it can be calculated for 442 nm and 532 nm excitation sources.

SR as a Function of Wavelength and Raman Shift

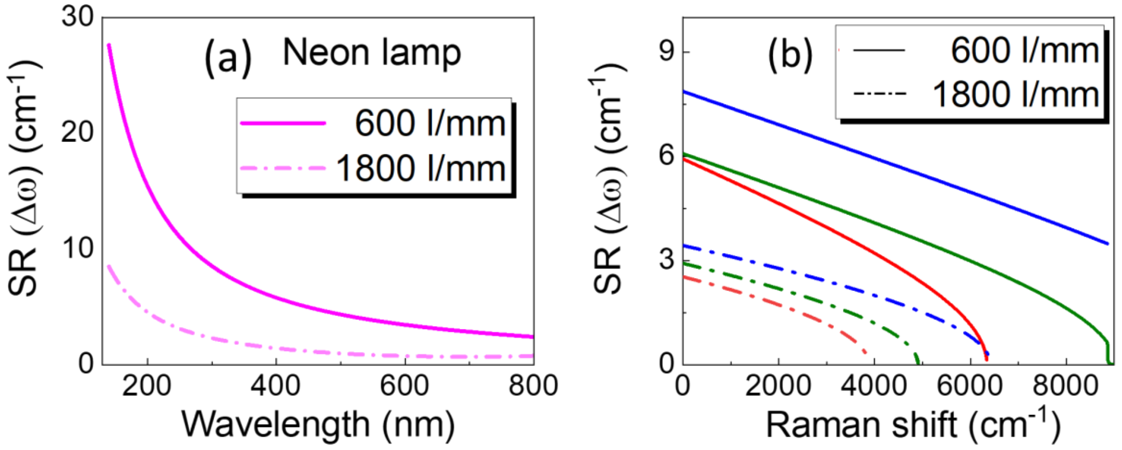

Figure S2. (a) SR as a function of wavelength for two available gratings 600 lines/mm and 1800 lines/mm and (b) SR as a function of Raman shift for two available gratings and three different excitation sources (442 nm, 532 nm, and 633 nm).

Figure S2a shows the dependence of SR as a function of wavelength for both gratings. This curve can be easily obtained from Eq. 1 by varying the wavelength (ω_L) over the desired range. Here, Q was kept constant (Q = 10 for 1800 l/mm and Q = 50 for 600 l/mm) that was originally obtained from experimental results as shown in Figure 2. Figure S2b shows the

dependence of SR as a function of Raman shift for two available gratings and three different excitation wavelengths (442 nm, 532 nm, and 633 nm).

Reference

1. E.G. Loewen, E. Popov. Diffraction Gratings and Applications. CRC Press, 2018.
